# Cystoid Macular Edema Associated With Venous Stasis in a Patient With Previously Undiagnosed Hyperhomocysteinemia

**DOI:** 10.7759/cureus.20782

**Published:** 2021-12-28

**Authors:** Stergios K Chaloulis, Konstantinos T Tsaousis

**Affiliations:** 1 Ophthalmology, Volos General Hospital, Volos, GRC

**Keywords:** retinal vein occlusion, oct angiography, hyperhomocysteinemia, venous stasis, macular edema

## Abstract

A 74-year-old female patient visited our department reporting unilateral painless vision reduction in her left eye noticed a few months ago. Clinical examination revealed decreased visual acuity in the left eye. Fundoscopy showed bilateral retinal venous stasis with cystoid macular edema in the affected eye, also confirmed by optical coherence tomography (OCT) imaging. OCT angiography showed no evidence of ischemia.

Bilateral findings raised suspicion for a systemic underlying cause. Laboratory tests showed elevated homocysteine plasma levels. Brain MRI showed ischemic encephalopathy.

Hyperhomocysteinemia creates a hypercoagulable state and therefore it is a risk factor for vascular thrombosis and retinal vein occlusion. Our patient was considered to suffer from an impending retinal vein occlusion due to venous stasis, causing a persistent macular edema, and, therefore, was treated with anti-vascular endothelial growth factor (VEGF) injections. She was also prescribed oral folic acid for life. Her visual acuity showed improvement and remained stable for a long period of time. When macular edema reoccurred she was treated with another intravitreal injection.

## Introduction

Retinal vein occlusion (RVO) is the second most common retinal vascular disease. Atherosclerosis plays an important role in the development of RVO. The retinal vein and artery share a common adventitial sheath at arteriovenous crossings so that atherosclerotic changes of the artery may compress the vein and precipitate retinal vein occlusion. It is often associated with systemic diseases. In many cases, it also occurs in young adults with no other systemic disease. Both local (raised intraocular pressure) and systemic risk factors (age, hypertension, diabetes mellitus, hyperlipidemia, smoking, hypercoagulable states of various etiologies, with hyperhomocysteinemia among them) have been associated with RVO [1, 2].

Based on the location of the occlusion, RVO is classified as central retinal vein occlusion (CRVO) or branch retinal vein occlusion (BRVO). Another classification of CRVO is non-ischemic and ischemic, with the latter having a worse visual prognosis and more severe complications [1].

There is also a type of incomplete vein occlusion characterized as impending CRVO, with rather mild symptoms and clinical findings. This may resolve or progress to complete vein obstruction [1]. Incomplete vein occlusion is also often referred to as venous stasis retinopathy.

Homocysteine (Hcys) is a sulfur-containing amino acid. All circulating Hcys are primarily derived by demethylation from dietary methionine. Abnormal rise of Hcys levels is characterized as hyperhomocysteinemia. [2]

The aim of our report is to present an interesting case of macular edema due to incomplete CRVO in a patient with increased levels of homocysteine.

## Case presentation

A 74-year-old female patient without past ocular history was referred to our department due to painless unilateral reduction of visual acuity in her left eye. The symptoms had been present for the last two months and were relatively stable. Her past medical history included arterial hypertension treated with angiotensin-converting enzyme (ACE) inhibitor and smoking. Despite her family medical history including diabetes (mother), she was not diabetic.

**Figure 1:**
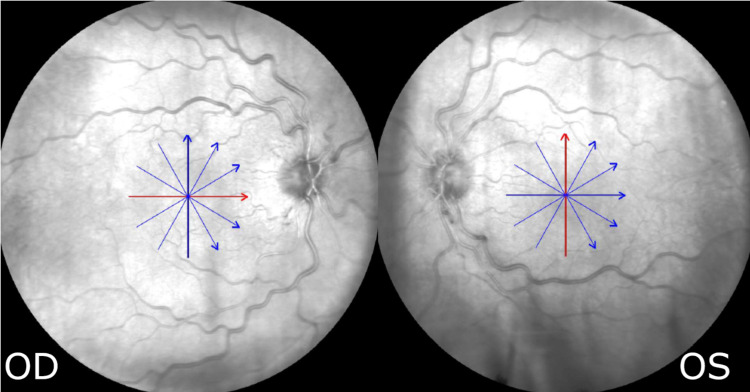
Fundus images. Scanning laser ophthalmoscopy (SLO) fundus images showing proportional marked vessel tortuosity in both eyes without other signs of complete vein occlusion (OD = Right eye, OS = Left eye)

At presentation, the patient’s best corrected visual acuity (BCVA) was 0.2 LogMAR in the right eye and 0.72 LogMAR in the left eye. Slit-lamp examination revealed mild nuclear sclerotic cataract in both eyes while intraocular pressure was within normal limits. Dilated fundoscopy revealed bilateral venous tortuosity without hemorrhages (Figure 1).

**Figure 2:**
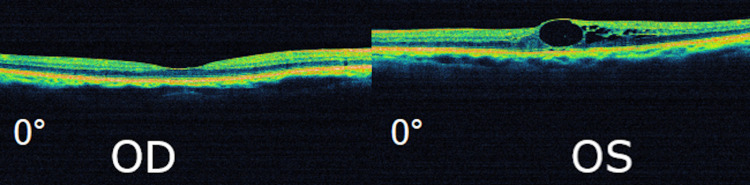
Initial OCT macular scan. OCT sections demonstrating significant cystoid macular edema only in the left eye (OD = Right eye, OS = Left eye, OCT = Optical coherence tomography)

Optical coherence tomography (OCT) imaging revealed normal anatomy for the right eye, however, it confirmed the presence of cystoid spaces in the macular area of the left eye (Figure 2).

**Figure 3:**
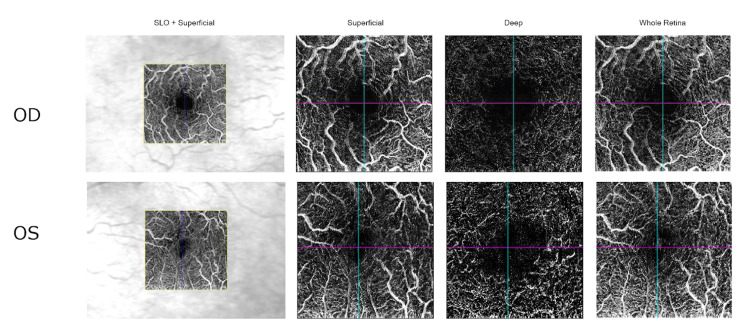
OCT angiography Optical coherence tomography angiography (OCT-A) images showing normal findings in the right eye and a suggestive appearance of cystoid spaces in the left eye (OD = Right eye, OS = Left eye)

Investigation with OCT angiography did not show any significant ischemic areas (Figure 3).

**Figure 4:**
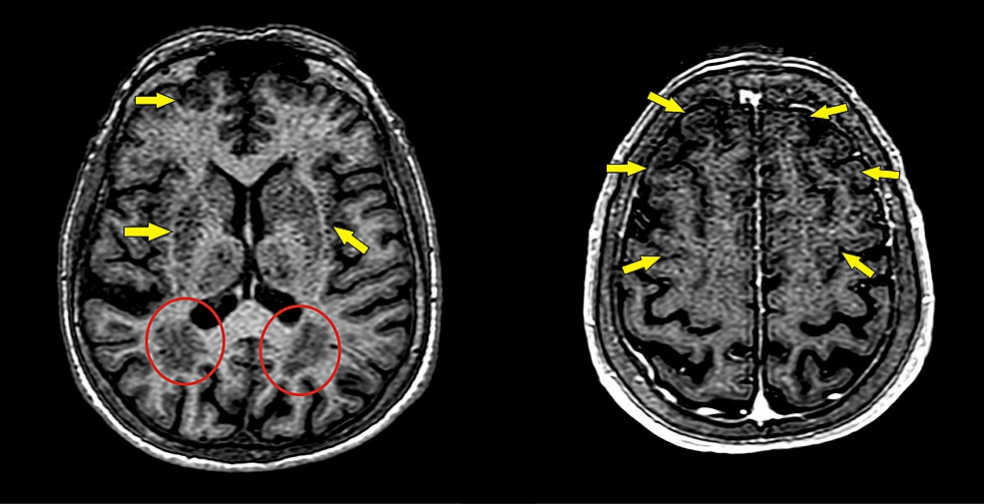
Brain MRI Brain 3D T1-weighted Magnetic Resonance Imaging with contrast media showing lesions of chronic cerebrovascular disease. Notice the areas of white matter hyperintensity (highlighted with red circles) and multiple lacunar infarcts in the thalamus and the frontal lobes (pointed with yellow arrows).

The bilateral venous stasis findings raised suspicion for a systemic cause of the condition and the macular edema. A thorough blood workup including coagulation markers has been ordered which revealed increased levels of homocysteine (23.3 μmol/L, laboratory normal range 5-13), as well as a mild increase of fibrinogen and fibronectin. Brain Magnetic Resonance Imaging (MRI) was also conducted. Radiologists reported no occlusion of major vessels but noted the presence of significant chronic ischemic encephalopathy (Figure 4).

The patient was referred to the haematologist who diagnosed hyperhomocysteinemia and prescribed oral folic acid for life. Regarding her ophthalmic treatment, a course of intravitreal injections with anti-vascular endothelial growth factor (VEGF) was scheduled for the left eye in order to remove the intraretinal fluid and improve visual acuity.

**Figure 5:**
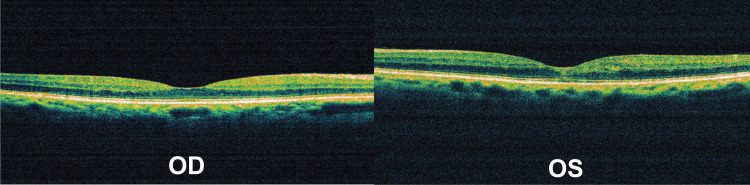
OCT four months post-treatment OCT sections showing a “dry” macula in the left eye, four months post-treatment with two intravitreal anti-VEGF injections (OCT = Optical coherence tomography, VEGF = vascular endothelial growth factor, OD = Right eye, OS = Left eye)

She was initially treated with two injections (one per month). Follow-up on a monthly basis showed an improved BCVA, reaching 0.52 LogMAR (further improving to 0.39 LogMAR with pinhole). Visual acuity remained stable to that level for a total of four months. Two consequent OCT scans during this period showed resolution of the macular cysts (Figure 5).

**Figure 6:**
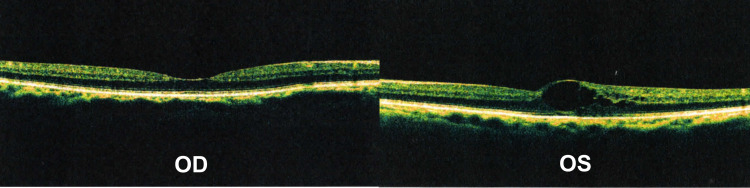
OCT six months post-treatment. OCT scan, six months post-treatment, showing recurrence of macular cyst in the left eye (OCT = Optical coherence tomography, OD = Right eye, OS = Left eye)

Based on these findings the decision was that no further treatment was necessary for the time being. A latter OCT scan, six months post-treatment, revealed recurrence of fluid accumulation in the macula of the affected eye without significant visual deterioration (Figure 6), therefore, the patient was treated again with intravitreal injection.

## Discussion

Impending CRVO is a relatively poorly-defined condition that, as mentioned above, can progress to complete CRVO. Typical clinical signs are mild venous dilatation and tortuosity with a few widely scattered flame-shaped haemorrhages. Fluorescein angiography (FA) assists diagnosis showing increased retinal circulation time. OCT facilitates monitoring the macular edema, when present [1]. The presented case resembles an impending central retinal vein occlusion whereas there were no ischemic areas in OCT angiography.

Mild to moderate increase of homocysteine plasma levels is a reported risk factor for atherosclerosis in the coronary, cerebral, and retinal vasculature [2]. This disorder may be due to an acquired nutritional deficiency in vitamin cofactors (folic acid, vitamins B6 and B12), or a chronic medical condition, or an adverse drug effect [2, 3]. Hyperhomocysteinemia is more frequently discovered in patients with RVO. [4]

Severe hyperhomocysteinemia (HH), on the other hand, is related to genetic mutations affecting the main two enzymes taking part in the metabolic pathway that converts homocysteine to other amino acids, methyltetrahydrofolate-homocysteine-methyltransferase (MTHFR) and cystathionine β-synthase. Homozygosity of the mutated allele results in greater severity hyperhomocysteinemia than heterozygosity. [3]

Hyperhomocysteinemia potentially caused the macular edema in our patient through two mechanisms. Firstly, indirectly, by causing elevation of venous and capillary pressure, stagnation of the blood flow (venous stasis) and subsequent hypoxia of the retina drained by the obstructed vein, damage to the capillaries and extravasations of blood elements [2]. Secondly, even in cases without complete vein occlusion, such as in the case presented here, by direct impairment of the inner blood-retinal barrier (BRB) [5]. Undoubtedly, smoking and hypertension were two additional risk factors, but the symmetricity of retinal appearance implies also a causal relation of the underlying hyperhomocysteinemia.

In cases of impending RVO, treatment is aimed at preventing progression to complete occlusion by correcting any predisposing systemic conditions and lowering intraocular pressure to improve perfusion [1]. Though there are well-established therapeutic protocols regarding anti-VEGF administration in macular edema due to complete RVO, there is no standard regimen for impending CRVO. In our patient, two loading doses of anti-VEGF resolved the macular edema. Further treatment was given following a “pro re nata” pattern. We also corrected hyperhomocysteinemia with oral folic acid.

## Conclusions

Bilateral retinal venous stasis with or without macular edema should raise suspicion of blood coagulability disorder, and relevant systemic workup could positively facilitate systemic management along with the required ophthalmic treatment. Also, when those findings involve a younger patient without vascular risk factors or co-morbidities, genotype investigation should be considered.
